# Busulfan for the treatment of myeloproliferative neoplasms: the Mayo Clinic experience

**DOI:** 10.1038/bcj.2016.34

**Published:** 2016-05-27

**Authors:** K Begna, A Abdelatif, S Schwager, C Hanson, A Pardanani, A Tefferi

**Affiliations:** 1 Division of Hematology, Department of Internal Medicine, Mayo Clinic, Rochester, MN, USA; 2 Department of Laboratory Medicine and Pathology, Mayo Clinic, Rochester, MN, USA

Busulfan (Myleran, Busulfex) is an alkylating agent that has been used for the treatment of myeloproliferative neoplasms (MPNs). It has been used since 1959, and it is a cell cycle non-specific alkylating agent. Busulfan is approved by the Food and Drug Administration and was commonly used for the treatment of chronic phase chronic myelogenous leukemia (a BCR-/ABL1-positive MPN) before it was displaced by the tyrosine kinase inhibitors (imatinib and other tyrosine kinase inhibitors). Busulfan has also shown significant activity in BCR-/ABL1-negative MPNs, as shown in a number of studies.^1, 2^ Similar to other alkylating agents (like chlorambucil), busulfan has been associated with an increased rate of leukemic transformation.^3, 4, 5, 6^ However, the particular studies were mostly retrospective and with small number of cases, and there is currently no controlled evidence to implicate busulfan as being leukemogenic in MPNs. Busulfan is currently used by many hematologists and oncologists as second-line treatment in patients with BCR-/ABL1-negative MPNs that are intolerant to or developed side effects from hydroxyurea. Furthermore, in a recently published study, use of busulfan in patients with polycythemia vera (PV) was accompanied by complete hematologic and molecular remission.^7^ In the current retrospective study, we wanted to share our experience with the use of busulfan in patients with BCR-/ABL1-negative MPN.

The current study was approved by the Mayo Clinic Institutional Review Board. Institutional databases from 1970 to 2014 were interrogated to identify informative patients by using the terms MPN, PV, essential thrombocythemia (ET), myelofibrosis (MF) and busulfan (or myleran). Patients' follow-up information was collected till July 2015. Seventy-five patients with full demographic, diagnostic and therapeutic information were identified: 37 patients with ET, 22 with PV, 12 with MF and 4 with MPN unclassifiable. Median age of the study population was 64 years (range 31–91). After a median follow-up of 17 years, 40 patients (53%) have died and leukemic transformation was documented in 4 (5%) patients with median time to leukemic transformation of 86 months (range 12–229).

Among 37 patients with ET, 29 (79%) were females and the median (range) age was 67 (33–90) years. At diagnosis, the median (range) hemoglobin (Hgb) (gm/dl), white blood cell count (WBC) (× 10^9^/l) and platelet count (× 10^9^/l) were 13.6 (9.8–16.9), 10.2 (5–231) and 1113 (593–2062), respectively. After a median follow-up time of 230 months, 15 patients (41%) have died and leukemic transformation was documented in only 1 patient who was also treated with radioactive phosphorous (P^32^). Leukemic transformation in the particular patient was documented 230 months from the date of diagnosis. Post-treatment, complete blood count was available in 20 patients and revealed median (range) Hgb, WBC and platelet count of 12 g/dl (9.9–16), 7.4 × 10^9^/l (3.1–25) and 267 × 10^9^/l (126–573), respectively.

Twenty-two patients with PV were identified, and 14 (61%) were females, and the median (range) age was 64 (46–91) years. At diagnosis, the median (range) Hgb, WBC and platelet count were 17.5 gm/dl (15.1–20.8), 11.5 × 10^9^/l (1.2–26.6) and 669 × 10^9^/l (185–2370), respectively. After a median follow-up time of 188 months, 13 (57%) patients have died, and leukemic transformation was documented in 2 patients, one of whom was also treated with P^32^. Post-treatment, complete blood count was available in 21 patients and revealed median (range) Hgb, WBC and platelet count of 12.9 gm/dl (10–15.2), 7.2 × 10^9^/l (2.8–20) and 303 × 10^9^/l (124–833), respectively.

Twelve patients with MF were identified. The median age was 52 years (31–75) and 5 were females. The median Hgb, WBC and platelet count were 13.6 gm/dl, 14.5 × 10^9^/l, and 472 × 10^9^/l. Six (50%) patients displayed splenomegaly and 5 of them have had splenic size reduction from 2–20 cm below the costal margin to 0–5 cm after busulfan treatment, 3 were splenectomized and 3 patients did not have palpable spleen. At a median follow-up of 208 months, and 39 months (range 78–401) from the start of busulfan treatment, 10 (84%) patients have died and no leukemic transformation was documented.

The preferred first-line cytoreductive therapy in patients with MPNs is hydroxyurea. Ruxolitinib (a JAK1/2 inhibitor) was recently approved for use in intermediate or high-risk treatment requiring patients with MF and hydroxyurea refractory/intolerant PV. The current study, as well as many other previously published ones, supports the use of busulfan as an alternative second-line therapy in MPNs, along with interferon-alpha, especially in ET and PV. However, some cautions need to be exercised while using busulfan in MPN. The drug can cause profound and prolonged cytopenias, especially thrombocytopenia. The dose of busulfan may need to be decreased when the platelet level drops below 300 × 10^9^/l, and the drug be held when the platelet counts drop below 150–200 × 10^9^/l.
